# Lipoprotein(a), homocysteine, and retinal arteriosclerosis

**Published:** 2008-09-15

**Authors:** Amir Ghorbanihaghjo, Alireza Javadzadeh, Hassan Argani, Nariman Nezami, Nadereh Rashtchizadeh, Mandana Rafeey, Mohammad Rohbaninoubar, Babak Rahimi-Ardabili

**Affiliations:** 1 Drug Applied Research Center, Tabriz University of Medical Sciences, Tabriz, Iran; 2 Biotechnology Research Center, Tabriz University of Medical Sciences, Tabriz, Iran; 3 Young Researchers Club, Tabriz, Iran; 4 Liver and Gastroenterology Research Center, Tabriz University of Medical Sciences, Tabriz, Iran

## Abstract

**Purpose:**

Elevated levels of lipoprotein(a) [Lp(a)] and homocysteine (Hcy) have been implicated as risk factors for vascular diseases. The study was performed to explore the possible relationship between retinal arteriosclerosis and serum Lp(a) and Hcy levels.

**Methods:**

Study subjects consisted of 80 nonsmoking male patients with retinal arteriosclerosis and 54 healthy nonsmoker males as controls. Retinal arteriosclerosis was graded according to the Scheie classification. Serum levels of lipids, lipoproteins, Lp(a), and Hcy were measured by standard methods.

**Results:**

The serum level of Hcy was higher in patients (24.2±8.1 μmol/l) than controls (10.5±4.1 μmol/l); p<0.01. Serum levels of Lp(a) in patients (47.9±33.1 mg/dl) was also higher than controls (11.7±7.6 mg/dl); p<0.01. There was a significant direct linear correlation between the degree of retinal arteriosclerosis and Lp(a) level (r=0.61, p<0.01), the degree of retinal arteriosclerosis and Hcy level (r=0.72, p<0.01), and also between Lp(a) and Hcy levels (r=0.67, p<0.01).

**Conclusions:**

The association between retinal arteriosclerosis and serum Lp(a) and Hcy levels suggests that Lp(a) as well as Hcy could play a role in the development of retinal arteriosclerosis.

## Introduction

Arteriosclerotic retinopathy involves vessel walls by medial layer hypertrophy, hyalinization in the intima, and hyperplasia in the endothelial layer [1]. Arteriosclerotic retinopathy usually occurs as a result of progressive hardening of blood vessels by calcification and loss of elastic tissue. Ophthalmoscopic examination is widely used in clinical practice to examine the presence and the degree of arteriolar retinopathy, and offers a unique noninvasive opportunity to assess the status of systemic arteriosclerosis [2,3]. The retinal arteriole shares similar anatomic and physiologic characteristics with cerebral and coronary microcirculation. Therefore, retinal microvascular disease may also reflect the presence of systemic microvascular disease [2,4]. Factors such as smoking, inflammation, and oxidative stress that are associated with free radical formation have been identified as possible risk factors for both systemic atherosclerosis and arteriolar retinopathy [5,6]. Hyperlipidemia is a powerful risk factor for atherosclerosis and related disorders such as ischemic heart disease, cerebrovascular diseases, and retinal atherosclerosis [7,8]. Although recent studies have shown that lipoprotein(a) [Lp(a)] and total homocysteine (Hcy) may be implicated in the development of systemic arteriosclerosis, little knowledge exists about their role in the pathophysiology of retinal arteriosclerosis [9-11].

Lp(a) particles contain the lipid and protein component of low-density lipoprotein (LDL) plus a glycoprotein known as apolipoprotein(a) [apo(a)] [12]. Apo(a) is homologous to plasminogen, and its similarity to plasminogen indicates a prothrombogenic role for Lp(a), whereas the similarity of Lp(a) to LDL suggests a proatherogenic role [13]. Serum Lp(a) concentrations are highly heritable. It is reported that about 90% of determinant factors are genetically dependent on sequences of the apo(a) gene in chromosome 6q26–27 [14,15]. Numerous studies have shown that serum levels of Lp(a) above 30 mg/dl is genetically determined and could be an independent risk factor for atherosclerotic vascular disease [16,17].

Both clinical and experimental investigations suggest that elevated fasting serum Hcy concentration also is an independent risk factor for atherosclerosis, including coronary artery disease, peripheral vascular disease, cerebrovascular disease, and venous thromboembolism [18,19]. Although the exact implication of increased Hcy on atherosclerosis is not clear, recent studies indicate that 15-30% of patients with premature occlusive vascular disease have moderately elevated Hcy concentrations (higher than 15 μmol/l) [19]. To date, little information exists on the possibility that elevated Hcy might increase the risk of retinal arteriosclerosis.

We proposed that elevated Lp(a) and Hcy levels synergistically increase the atherogenic process, as the potential biochemical interactions with the two risk factors have already been reported [11,20].

Although Lp(a) and Hcy levels are higher in systemic atherosclerosis, their relationship with retinal arteriosclerosis still is not clear [21-23]. The aim of this study was to assess the serum concentration of lipids and lipoproteins as well as investigate the concentrations of Hcy and Lp(a) and their relationship with retinal arteriosclerosis.

## Methods

This case-control study was performed from July, 2005 to May, 2006 in the Retina service, Department of Ophthalmology of Tabriz University of Medical Sciences. All participants were recruited from male patients presenting for routine eye examination at the Tabriz Nikookari Eye Hospital. Females were excluded from the study as it has been hypothesized that there is a close relationship between serum estrogen and Hcy levels [24,25]; in addition, the influence of sex hormones on Hcy concentration is not conclusive [26-29]. The ethic committee at Tabriz University of Medical Sciences reviewed and approved the present study which is in compliance with the Declaration of Helsinki.

This study enrolled 80 men with retinal arteriosclerosis and a control group of 54 healthy males. All participants gave informed consent. Each participant was evaluated by a retinal specialist (A.J.). Retinal arteriosclerosis was confirmed by slit-lamp examination with super-field lens and fundus photography (Image net 2000, Topcon TRC50IX, Topcon Corp, Tokyo, Japan). Retinal arteriosclerosis was graded according to the Scheie classification for arteriosclerosis (Table 1) [30,31]. In addition to female patients, also excluded were patients who were being treated with vitamins, antioxidants, micronutrients supplements, or lipid-lowering drugs; patients who had active systemic infection; smokers; and patients who had a history of alcohol abuse, coronary heart diseases, uncontrolled hypertension (systolic blood pressure ≥140 mmHg, diastolic blood pressure ≥90 mmHg), diabetes mellitus, thyroid, or other metabolic diseases. All participants had normal renal and liver functions as assessed by plasma urea, creatinine, alanine aminotransferase, and aspartate aminotransferase. Blood samples were obtained after an overnight fasting. Serum samples were frozen immediately and stored at -70 °C until needed for analysis. Serum levels of total cholesterol (TC), triglyceride (TG), high-density lipoprotein cholesterol (HDL-C) were determined using commercial reagents with an automated chemical analyzer (Abbott analyzer, Abbott laboratories, Abbott Park, North Chicago, IL). Low-density lipoprotein cholesterol (LDL-C) was calculated by using the Friedewald equation [32]. The serum level of Lp(a) was assayed by commercially available immunoturbidimetric kit (Pars Azmun, Tehran, Iran, Lott No: 85001) using the same analyzer. Serum Hcy concentration was determined by a commercially available enzyme-linked immunoassay (Axis-Shield, Axis Biochemicals ASA, Distributed by IBL, Hamburg, Germany, Cat. No: AX51301).

**Table 1:** Scheie classification

**Stage**	**Observation**
0	Normal
1	There is broadening of the light reflex from the arteriole, with minimal or no arteriolovenous compression.
2	Light reflex changes and crossing changes are more prominent.
3	The arterioles have a copper wire appearance, and there is more arteriolovenous compression.
4	The arterioles have a silver wire appearance, and the arteriolovenous crossing changes are most severe.

Retinal arteriosclerosis was graded by slit-lamp with super-field lens and fundus photography in retina according to Scheie classification as above [30,31].

SPSS software package version 13 for Windows (SPSS Ins, Chicago, IL) was used to perform statistical analysis. Results were expressed as mean ± standard deviation. Independent *t*-test, Mann–Whitney U, logistic and multiple regression tests, as appropriate, were used to assess significance of differences between the two groups. The correlations between variables were evaluated by Pearson (for parametric data) or Spearman (for nonparametric data) test as appropriate. A p<0.05 was considered significance statistically.

## Results

We compared a group of 80 male patients with retinal arteriosclerosis to 54 male control patients. There were no statistically significant differences between the mean ages of case (64.3±6.8 years) and control (66.7±8.0 years) groups (p>0.05). There was no significant difference in systolic blood pressure between the case (116.90±10.47 mmHg) and control groups (113.55±10.12 mmHg); p>0.05. Also diastolic blood pressure in the case group (69.81±8.50 mmHg) was not statistically different from control group (67.85±8.05 mmHg); p>0.05.

All controls had degree 0 of retinal arteriosclerosis (by slit-lamp with super-field lens and fundus photography in retina according to Scheie classification in Table 1), while 24 (30%) of patients were affected with degree I, 21 (26.25%) with degree II, 21 (26.25%) with degree III, and 14 (17.5%) with degree IV.

Lipid profile of patients and control group are shown in Table 2. There were no differences in HDL levels between the two groups, while the mean levels of TG, TC, and LDL were significantly higher in patients than those in control group. Serum level of Hcy was higher in patients (24.2±8.1 μmol/l) than controls (10.5±4.1 μmol/l); p<0.01. Serum levels of Lp(a) in patients (47.9±33.1 mg/dl) was also higher than controls (11.7±7.6 mg/dl); p<0.01.

**Table 2:** Lipid profiles of patients with retinal arteriosclerosis and controls

**Variables**	**Controls (n=54) mean±SD**	**Patients (n=80) mean±SD**	**p value**
TC (mg/dl)	159.4±25.2	199.8±39.6	<0.0001*
TG (mg/dl)	114.8±35.3	152.4±71.8	0.001**
HDL-C (mg/dl)	37.4±9.5	39.7±8.6	>0.1*
LDL-C (mg/dl)	99.5±22.9	129.6±37.8	<0.0001*

This table shows the comparison of the lipid profiles between the patients and controls. Except the high-density lipoprotein cholesterol (HDL-C) level, all the total cholesterol (TC), triglyceride (TG), and low-density lipoprotein cholesterol (LDL-C) levels are higher in patients than controls. The asterisk denotes an independent-sample *t*-test, and the double asterisk indicates a Mann-Whitney U test.

**Figure 1:**
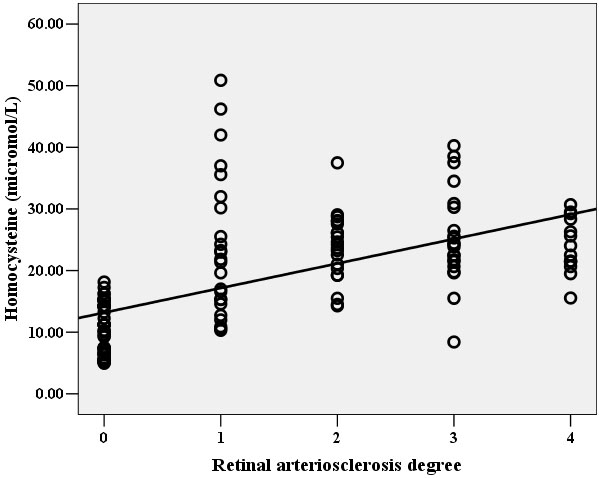
Relationship between Hcy level and the degree of retinal arteriosclerosis. There was a direct linear correlation between Hcy level and the degree of retinal arteriosclerosis in the study population (r=0.72, p<0.01), i.e. patients with higher degree of retinal arteriosclerosis had higher level of Hcy.

**Figure 2:**
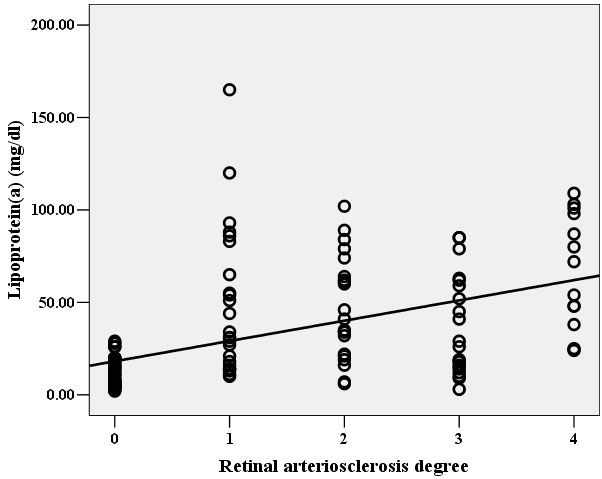
Relationship between Lp(a) level and the degree of retinal arteriosclerosis. There was a direct linear correlation between Lp(a) level and the degree of retinal arteriosclerosis in the study population (r=0.61, p<0.01), i.e. patients with higher degree of retinal arteriosclerosis had higher level of Lp(a)

**Figure 3:**
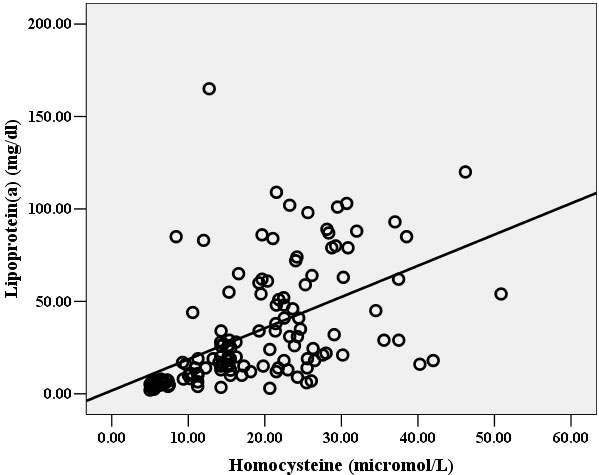
Correlation of Hcy level with Lp(a) level. There was a direct linear correlation between Hcy and Lp(a) levels in the study population (r=0.67, p<0.01), i.e., higher level of Hcy was associated with higher level of Lp(a).

Hcy and Lp(a) levels were both significantly correlated with the degree of retinal arteriosclerosis (Figure 1 and Figure 2). In multivariable analysis, retinal arteriosclerosis was associated with higher levels of Lp(a) (OR: 1.13, 95% CI: 1.07-1.19; P<0.01) and Hcy (OR: 1.52; 95% CI: 1.31-1.77; p<0.001). There was no statistically significant association of retinal arteriosclerosis with age, systolic and diastolic blood pressure, TC, TG, LDL-C, and HDL-C. However, there was a significant direct linear correlation between Lp(a) and Hcy (Figure 3).

**Figure 4:**
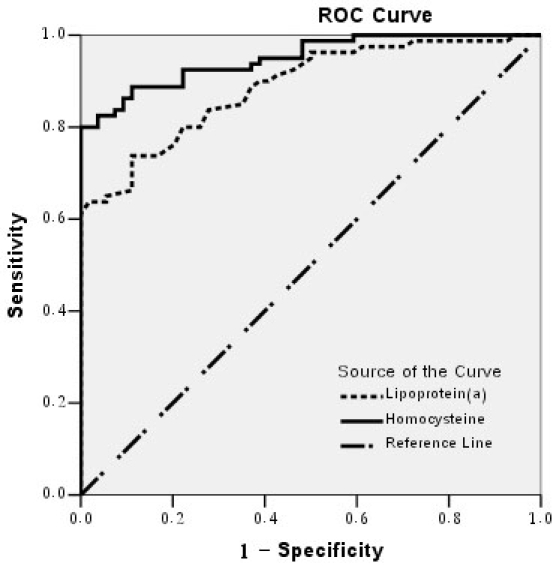
Receiver operating characteristic curves for arteriosclerosis. Shown is the relationship of sensitivity to 1-specificity as plotted for lipoprotein(a) and homocysteine. The area under each receiver operating characteristic (ROC) curve for each parameter indicates its diagnostic accuracy (Table 3).

Consistent with previous reports [20,33], 30 mg/dl of Lp(a) (likelihood ratio= 69.11, P<0.0001) and 15.5 μmol/l of Hcy (likelihood ratio= 86.93, p<0.0001) were determined as significant cutoff points for arteriosclerosis. Lp(a) and Hcy performance as a predictor of arteriosclerosis was summarized by receiver operating characteristic (ROC) curve (Figure 4) and area under the curve (AUC; Table 3). ROC curve was well above the diagonal, indicating good sensitivity and specificity. The AUC indicated a high probability of correctly prediction of arteriosclerosis.

**Table 3:** Area under the curve for the receiver operating characteristic curves in Figure 4

**Test result variables**	**Area under curve**	**Standard error**	**Asymptotic sig.**	**Asymptotic 95% confidence interval**	
				**Lower bound**	**Upper bound**
Lipoprotein(a)	0.888	0.027	0.000	0.835	0.941
Homocysteine	0.950	0.017	0.000	0.917	0.983

This table shows the values of area under the curve for the receiver operating characteristic curves of lipoprotein (a) and homocysteine in Figure 4.

## Discussion

Increased blood pressure, elevated total cholesterol, smoking status, coronary bypass surgery history, and high white blood cell count are important etiologic factors in retinal arteriosclerosis, but there is limited information about a possible role of Lp(a) and Hcy in the pathophysiology of retinal arteriosclerosis [1,4,19]. It is well known that elevated levels of Hcy and Lp(a) are often associated with endothelial dysfunction and enhanced atherosclerosis [12,34]. Various authors have proposed that hyperhomocysteinemia and high serum Lp(a) concentrations play a role in the genesis of systemic atherosclerosis, thrombosis, and related disorders [11,20,35,36].

The retina is the only place where we can directly observe arteriosclerotic changes noninvasively [1-3]. Since both cerebral and retinal arteries are peripheral branches of the internal carotid arteries, high Lp(a) and Hcy levels may also be associated with retinal vascular diseases such as retinal arteriosclerosis [2,4,37]. To evaluate the connection between Lp(a) and Hcy levels and retinal arteriosclerosis, we determined serum Lp(a) and Hcy concentrations and lipid and lipoprotein levels. In the present study, patients had significantly higher Hcy and Lp(a) levels. These results are in agreement with those reported by Martin et al., [38] who suggested Hcy might be a risk factor in retinal vascular disease. Present study findings suggest that hyperhomocysteinemia may play an important role in the pathogenesis of retinal arteriosclerosis.

Lp(a) is a low-density lipoprotein particle in which apolipoprotein B-100 is linked by a single interchain disulfide bridge to a unique glycoprotein apo(a) [39,40]. It has been shown that high plasma Lp(a) levels are closely associated with arterial thrombosis such as myocardial infarction and cerebral infarction [16,41,42]. The role of Lp(a) in retinal vascular changes has been reported in a few cases [43-46]. Some investigators have proposed that impaired fibrinolysis and atherogenesis induced by Lp(a) may play a role in the pathophysiology of retinal vascular changes, while other investigators have concluded that serum Lp(a) level is not a significant risk factor for atherosclerosis of the retinal arteries [44,45]. Foody et al. [20] found that thiols, such as Hcy, can dissociate apo(a) from Lp(a) complex, leading to the exposure of an additional lysine binding site on apo(a) that can increase the affinity of apo(a) to plasmin-modified fibrin. In the present study, correlations between Lp(a), Hcy, and the degree of retinal arteriosclerosis were also investigated. The data indicate that there is a significant correlation between the degree of retinal arteriosclerosis with both Lp(a) and Hcy levels. Moreover, a significant correlation between Lp(a) and Hcy concentrations was observed. However, because this study only included male participants and the study population was of a low number, further studies are warranted.

In conclusion, our findings support the idea that Lp(a) and Hcy may play an important role in the development of retinal arteriosclerosis.
